# Risk assessment of lead and cadmium concentrations in hen's eggs using Monte Carlo simulations

**DOI:** 10.1002/fsn3.3268

**Published:** 2023-03-08

**Authors:** Hedayat Hoseini, Abdol‐Samad Abedi, Fatemeh Mohammadi‐Nasrabadi, Yeganeh Salmani, Fatemeh Esfarjani

**Affiliations:** ^1^ Department of Food Science and Technology, Faculty of Nutrition Sciences and Food Technology, National Nutrition and Food Technology Research Institute Shahid Beheshti University of Medical Sciences Tehran Iran; ^2^ Food and Nutrition Policy and Planning Research Department, Faculty of Nutrition Sciences and Food Technology, National Nutrition and Food Technology Research Institute Shahid Beheshti University of Medical Sciences Tehran Iran

**Keywords:** cadmium, health risk assessment, heavy metal, hen egg, lead, simulation

## Abstract

The hen egg is one of the main sources of cheap, great quality, and nutritious food. This study aimed at determining the level of lead (Pb) and cadmium (Cd) in hen eggs and at assessing the carcinogenic and non‐carcinogenic risks caused by the consumption of hen eggs collected in Iran. A total of 42 hen eggs from 17 major brands were randomly sampled from supermarkets. Lead and cadmium concentrations were determined by using inductively coupled plasma mass spectrometry (ICP‐MS). Additionally, using the Monte Carlo simulation (MCS) method to calculate dietary exposure, target hazard quotient (THQ), and incremental lifetime cancer risk (ILCR), the related human health risk associated with ingesting these hazardous metals for adults was evaluated. The average Pb and Cd concentrations in whole eggs were 7.16 ± 0.248, and 2.83 ± 0.151 μg kg^−1^, respectively, which were less than the maximum permitted levels, established by FAO/WHO and the Institute of Standards and Industrial Research of Iran (ISIRI). Pb and Cd concentrations were significantly correlated at the 0.05 level (*r* = 0.350). Regarding the levels of Pb and Cd in eggs, overall, the estimated weekly intake (EWI) of these metals for adults by egg consumption was determined 0.014 and 0.007 mg/week, respectively, which were lower than the risk values suggested. The carcinogenic and non‐carcinogenic indexes of Cd and Pb indicated that the adult population in Iran was safe (THQ Pb and Cd <1, ILCR <10^−6^ Pb). It should be emphasized that this research primarily focuses on egg consumption, which may account for a relatively small portion of Iranian consumers' overall exposure to Pb and Cd. Therefore, a comprehensive study on the risk assessment of these metals through whole‐diet foods is recommended. The findings showed that lead and cadmium levels in all evaluated eggs were suitable for human consumption. Adults' Pb and Cd exposure from eating eggs was significantly lower than the risk levels established by Joint FAO/WHO Expert Committee on Food Additives (JECFA), per the exposure assessment. According to the THQ values of these dangerous metals, which were below one value, egg eating by Iranian consumers does not present a non‐carcinogenic risk. In addition, this finding provides accurate and reliable information for policymakers to improve food safety status to reduce public health hazards.

## Graphical abstract

Consumers were not at significant non‐carcinogenic risk of Pb and Cd (THQ ≤1). Consumers were at threshold carcinogenic risk of Pb (ILCR >10^−4^).

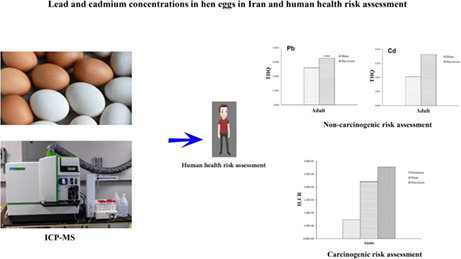

## 1 INTRODUCTION

One of the primary sources of affordable, high‐quality, and nutrient‐dense protein with a variety of mineral salts and vitamins is the hen egg (Talakesh et al., 2020). The World Health Organization (WHO) states that hen eggs are an essential part of the human diet, but that their consumption is higher than that of other sources of animal proteins because of economic issues (Fakhri et al., 2018; Talakesh et al., 2020). The Food and Agriculture Organization (FAO) predicts that egg consumption would rise from 6.5 to 8.9 kg per year in impoverished countries to 13.5 to 13.8 kg per year in industrialized countries (Talakesh et al., 2020). *The World Health Organization* (WHO) recommended that for a requirement of 2100 kcal consume 60 g of eggs on average twice or 4 times a week (Giannetto et al., 2016) The average annual per capita consumption of hen eggs in the world is 250 Kg. However, per capita, egg consumption among households in Tehran is much lower than the recommended standards (Talakesh et al., 2020). Iranians consume about 198 kg of eggs annually, despite being the eleventh‐largest egg producer in the world (Fakhri et al., 2018).

Either during production or consumption, metals can taint eggs. Eggs may become tainted with heavy metals through chicken feed and drinking water, both of which are largely influenced by the environment. Heavy metals have several acute and long‐term harmful effects on different human organs. Examples of the adverse effects of heavy metal toxicity include cancer, gastrointestinal and renal dysfunction, nervous system diseases, skin lesions, vascular damage, immune system malfunction, and birth abnormalities (Balali‐Mood et al., 2021; Johri et al., 2010).

Heavy metal contamination of eggs is a significant problem for public health. Long‐term exposure to even small doses may cause anxiety, depression, restlessness, hypertension, anemia, damage to the fetal brain, tremors, lungs, liver, kidney diseases, and autoimmunity diseases, such as rheumatoid arthritis (Aliu et al., 2021; Järup, 2003). According to studies, low‐dose exposure to Cd, and Pb, interactions in mixtures showed higher levels of toxicity than individual heavy metals (Cobbina et al., 2015). Exposure to Pb has been linked to gastrointestinal colitis, brain function disorders, anemia, leukemia, hyperactivity, and thrombotic diseases (Abedi et al., 2020). Additionally, substances containing cadmium and lead were identified as carcinogens to humans. Exposure to Cd is related to painful diseases known as Itai‐Itai. Hypercalciuria, inconsistent calcium metabolism, hypertension in expectant women, kidney stone formation, and bone demineralization are some further negative impacts of Cd on human health. (Satarug et al., 2017). Due to the extensive use of pesticides, municipal wastewater, industrial effluents, and raw sewage for irrigation, both necessary and nonessential components are continually replenishing our food chain (Mitra et al., 2022). One of the major problems posed by heavy metals is that they are not usually metabolized by the body (Wang et al., 2017). Tehran, the capital of Iran, has received a reputation as one of the world's most polluted cities in recent years (Goharipour & Firoozabadi, 2022). This pollution can affect human health directly or indirectly through contamination of the food chain. The food system depends heavily on chickens, and their contamination can have disastrous effects. Therefore, this study was conducted with a special look at hen eggs as one of the most consumed foods in the food basket of most low‐income Iranian households. Pb and Cd concentrations, which are the two most hazardous heavy metals due to their cumulatively harmful influence on human health, were used to evaluate the quality of eggs to determine their potential risk to human health. Because hen eggs are one of the most popular foods in the food baskets of the majority of low‐income Iranian households. Egg quality was assessed to establish the possible risk to human health using Pb and Cd concentrations, the two most dangerous heavy metals due to their cumulatively negative impact on human health.

## 2 MATERIALS AND METHODS

### 2.1 Samples

Forty‐two egg samples were selected from 21 best‐selling brands of supermarkets in Tehran. All samples (two eggs from each batch content of 12 eggs) were first visually inspected by candling and only fresh and unscathed eggs (no cracks, visibly clean eggs, and pin‐holes) were chosen. Then, they were cleaned with distilled water and after coding and transferred to the laboratory for chemical analysis. There, samples were immediately refrigerated at 4°C before the preparation process. Instead of using metal tools, chemically stable sterile flacon tube tools were utilized to prevent any chemical contamination. The preparation for the analytical work was done right away. The entire piece of equipment was cleaned with diluted Nitric acid (HNO_3_10%) and then distilled water before use to prevent element contamination (Meermann & Nischwitz, 2018).

### 2.2 Samples preparation

Before being weighed with a computerized analytical balance that had a 0.0001 g precision, all samples were thoroughly cleaned in deionized water. The eggshell is then gently separated from the egg's contents. The next step involved carefully mixing and homogenizing each complete egg product before pouring it into Petri plates to dry for 24 h at 70°C.

To digest half a gram of dried egg samples, 10 mL of 70% nitric acid and 30% hydrogen peroxide (v/v) were purchased from Merk (Darmstadt, Germany) and left at room temperature for one night. Until the solution was clear, the digestion was finished at 150°C for 4 h after the solution had been cooled to room temperature (22–23°C), it was diluted to 50 mL with deiodine water and then filtered through 0.45 L of acid‐resistant filter paper. For further analysis, the solution was kept at 4°C. The liquefied solution was filtrated and diluted with 20% HNO_3_ before being analyzed by inductively coupled plasma mass spectrometry (ICP‐MS, ULTIMA2, 6100 DRC‐e Perkin Elmer Elan). The following steps were done: washing glassware containers used for analysis with detergent and rinsing several times with tap water, soaking overnight in 6 N HNO_3_ (Merk) solutions, and finally rinsing with deionized water.

According to the FDA Elemental Analysis Manual, samples were examined for total Pb and Cd using heat‐block‐assisted acid digestion and the ICP‐MS technique. The blank solution was made in the same way but without an egg. In Table 1, the ICP‐MS conditions for detecting lead and cadmium in eggs are listed ICP‐MS.

**TABLE 1:** Conditions of ICP‐MS apparatus for determining lead and cadmium in eggs.

Parameter	Value
Radiofrequency	1200 W (40 Mhz)
Plasma gas (Argon) flow	16 L/min
Nebulizer gas (Argon) flow	1 L/min
Read delay and analysis speeding	30 s
Wash	60 s
Wash speeding	30 rpm
Dwell time	50 ms
Resulting/amu 10% peak	0.7
Integration time	3.5
Linear working range (total element) ppb	0.053
Precision %RSD *n* = 10	1.3
Addition/recovery	93–103
Repetition	3

Ionizing the sample with an inductively coupled plasma is the method used in inductively coupled plasma mass spectrometry or ICP‐MS. It is well known for its application in identifying a variety of non‐metals and metals in liquid samples at incredibly low concentrations. It is well known that this method is the most rapid and accurate for detecting heavy metal concentration in the food industry (Meermann & Nischwitz, 2018).

### 2.3 Health risk assessment

#### 2.3.1 Estimate daily intake

The average body weight (BW) of adult consumers, the mean levels of these metals in eggs, and the number of eggs ingested were used to compute the Estimate daily intake (EDI) of Pb and Cd.

The EDI of Pb and Cd was estimated according to Equation 1 (Dadar et al., 2017).

(1) $$\mathrm{EDI}=F_{\mathrm{IR}}\times C_{M}/W_{\mathrm{AB}}$$

Estimate daily intake is estimated daily intake (μg kg^−1^ BW/day); F_IR_, daily eggs consumption (ml day^−1^); C_M_, mean level of metal (mg mL^−1^); and W_AB_, and average body weight (kg). According to the report of the National Nutrition and Food Technology Research Institute (NNFTRI, 2022), the number of eggs consumed by adults (18–50) in Iran is 25.4 g/day. In addition, the EPA's body weight studies indicate that the WAB (body weight) for adults is 70 (Environmental Protection Agency (EPA), 2011; Phillips & Moya, 2014). The exposure of lead and cadmium by consuming eggs was detected based on two situations of the highest and overall concentrations of these metals. Moreover for EDI, the estimated weekly intake (EWI, μg kg^−1^ BW/week) of Pd and Cd and also estimated monthly intake (EMI, μg kg^−1^ BW/month) of cadmium for adults were calculated to compare with the PTWI (provisional tolerable weekly intake) and PTMI (provisional tolerable monthly intake) set by (Joint FAO/WHO Expert Committee on Food Additives (JECFA), 2011).

### 2.4 Non‐carcinogenic risk estimation

The non‐carcinogenic danger to egg consumers was assessed using the target hazard quotient (THQ) of Pb and Cd from hen eggs (EPA, 2004; Rahmani et al., 2018) by equation 2:

(2) THQ=EF×ED×FIR×CM/RFD×WAB×TA

The frequency of exposure (EF) and exposure time (EP) deemed necessary to achieve the Iranian mean longevity were 365 days annually and 70 years, respectively. Pb and Cd had oral reference doses (RFD) of 0.004 and 0.001, respectively. For non‐carcinogens, the TA was 25,550 days (EF × ED).

### 2.5 Carcinogenic risk estimation

The incremental lifetime cancer risk (ILCR) was estimated to determine the possibility of cancer risk of Pb in adults through consuming eggs (Abedi et al., 2020) and calculated by equation 3:

(3) ILCR=EDI×CSF

The cancer slope factor (CSF) is the risk produced by a lifetime mean dose of 1 mg kg^−1^ BW/day (Fakhri et al., 2018). CSF for Pb was 0.0085 mg kg^−1^ day (OEHHA, 2009).

### 2.6 Monte Carlo simulation (MCS) technique

Estimating health risks can involve some uncertainty (Chen et al., 2012). High uncertainty is seen when using single‐point measurements to determine health risks associated with exposure to contaminants such as hazardous metals. To reduce the uncertainties in the health risk assessment, MCS was employed in our investigation as a probabilistic technique (Keramati et al., 2018; Ru et al., 2013). The creation of risk assessment models was done using the Crystal Ball software (version 11.1.2.4, Oracle, Inc., USA). The criteria for endangered exposed populations is a percentile of 95% of THQ and ILCR in the cumulative probability graph, and there were 1000 repeats. (Qu et al., 2012).

### 2.7 Statistical analysis

Data analysis was carried out by the SPSS software (SPSS Inc., version 16, Chicago, IL, USA) following the collection of the required data. For categorical variables, data were summarized using frequency (%), mean (±Standard Error), and median (minimum‐maximum) for both normal and non‐normal distributions. The data's normality was examined using the Kolmogorov–Smirnov test. Kruskal–Wallis and Spearman's correlation matrix analyses were used to compare two groups against one another. *p*‐values <.05 were considered statistically significant.

The average of two measurements, with repeatability of under 10%, was used to determine the trace element concentrations in each sample. The results were given in milligrams per kilogram of the sample's moist weight. For the statistical analysis, it was assumed that the concentration was equal to LOQ/2 when the element's content was below the limit of quantification (LOQ) (Esposito et al., 2016).

## 3 RESULTS AND DISCUSSION

Heavy metal exposure has grown as a result of environmental, industrial, and agricultural activity as well as modern industrialization, all of which have negative impacts on human health. The environmental problem of hazardous metal contamination of water and air affects hundreds of millions of people worldwide. Heavy metal pollution in food is a problem for both human and animal health. In this context, the concentration of heavy metals in food, air, and water resources is evaluated (Luo et al., 2021). The hazardous metal lead has a significant negative influence on individuals and communities. It can impact psychological and behavioral functions when present in low concentrations, and it can be harmful when present in high quantities (Tong et al., 2000). Exposure to Pb may raise the risk of stomach, kidney, lung, and brain cancer in both men and women (Wang et al., 2021).

In nature, lead is a nonbiodegradable metal that occurs in quite small concentrations. Human activities including manufacturing, mining, and the combustion of fossil fuels are causing atmospheric lead levels to constantly rise. When exposure levels are higher than what is considered safe, lead is harmful to humans. Children are more likely to become poisoned by lead than adults, and the severity of the poisoning rises when they come into touch with dust that contains environmental lead (Luo et al., 2021). And also, cadmium is a toxicant and carcinogenic metal. In addition to its carcinogenic properties, cadmium induces kidney disease, bone disease, and cardiovascular disease. Low‐to‐moderate cadmium exposure results in hypertension, diabetes, carotid atherosclerosis, peripheral arterial disease, chronic kidney disease, myocardial infarction, stroke, and heart failure (Tellez‐Plaza et al., 2012).

The operating limitations for ICP‐MS trace element determination were investigated. Pb and Cd values in hen eggs were 7.158 ± 0.240 and 2.83 ± 0.151 (μg kg^−1^), respectively, on average. Pb and Cd levels obtained from the analyses of all eggs samples were lower than 500 and 50 μg kg^−1^, the limits as established by the FAO/WHO (Kabeer et al., 2021; ul Islam et al., 2014). The correlation between the Pb and Cd was significant at the 0.05 level (*r* = 0.350) in Table 2.

**TABLE 2:** Concentration of Pb and Cd (μg kg^−1^) in hen eggs.

Variables	Range	Min.	Max.	Mean ± SE
Pb	6.6	2.400	9.000	7.158 ± 0.248
Cd	3.81	1.190	5.000	2.83 ± 0.151

*Note*: The correlation between the Pb and Cd was significant at the 0.05 level (0.350).

The typical level of lead found in hen eggs in this study and other previous studies in Turkey (Uluozlu et al., 2009), China (Zheng et al., 2007), England (Ysart et al., 2000), and Canada (Kirkpatrick & Coffin, 1975) was below the standard limits; however, some studies in Iran (Farahani et al., 2015; Salar‐Amoli & Ali‐Esfahani, 2015) and in India (Basha et al., 2013), Pakistan (Khan & Naeem, 2006), and Nigeria (Fakayode & Olu‐Owolabi, 2003) have found above limited amounts. Two other studies in the USA and a study in Palestine reported lead concentrations above 0.97, 0.167, and 0.27 mg kg^−1^, respectively (Abdulkhaliq et al., 2012; Bautista et al., 2014; Spliethoff et al., 2014; Table 3).

**TABLE 3:** Comparison of the heavy metals concentration (mg kg^−1^) with other studies.

Study	Country	Pb	Cd	Ref.
Abbasi Kia et al. (2017)	Iran	0.074	0.01	Abbasi Kia et al. (2017)
Farahani et al. (2015)	Iran	0.75	0.248	Farahani et al. (2015)
Faryabi et al. (2021)	Iran	0.09	–	Faryabi et al. (2021)
Arslanbaş and Baydan (2013)	Turkey	0.05	2.33	Arslanbaş and Baydan (2013)
Khan et al. (2015)	Pakistan	0.13	0.075	Khan et al. (2015)
Zheng et al. (2007)	China	0.052	0.002	Zheng et al. (2007)
Giri and Singh (2019)	India	0.51	0.008	Giri and Singh (2019)
Ysart et al. (2000)	UK	0.003	–	Ysart et al. (2000)
Current study		0.007	0.002	–

*Note*: ISIRI (Institute of Standards and Industrial Research of Iran) and FAO/WHO (2002) and codex Alimentations allowed limits: Pb = 0.1, Cd = 0.05 mg kg^−1^.

According to this study, cadmium levels in hen eggs are on par with those in England. (Ysart et al., 2000), India (Basha et al., 2013), China (Zheng et al., 2007), and some other studies such as Nigeria (Fakayode & Olu‐Owolabi, 2003), Canada (Kirkpatrick & Coffin, 1975), Pakistan (Khan & Naeem, 2006), and Iran (44) although were upper than those studies, they were still under the limitation. Two studies in Iran (Farahani et al., 2015; Salar‐Amoli & Ali‐Esfahani, 2015) and a study in Turkey (Uluozlu et al., 2009) were above the cadmium limits. (Table 3).

According to Iranian research, there is no need for intervention or remediation to lessen the burden of heavy metal contamination in these poultry sectors because the health risks of the heavy metals examined in Iranian egg products, whether they are cancerous or not, are safe for a variety of consumers. (Abbasi Kia et al., 2017).

### 3.1 Health risk assessment

In addition to estimating the overall concentration of lead and cadmium by comparing the standard permissible limits, other parameters like exposure time, per capita intake, metal toxicity, and body weight are highly useful in determining the potential health risk. Therefore, exposure assessments as well as non‐carcinogenic and carcinogenic risk assessments for adults were carried out using accurate data interpretation (Fakhri et al., 2018; Rahmani et al., 2018).

### 3.2 Exposure assessment

Estimate daily intake, EWI, and EMI were calculated using two scenarios for the total and maximum concentrations of these metals, and the findings were compared with the JECFA‐set provisional acceptable weekly intake (PTWI) and provisional tolerable monthly intake (PTMI); it was possible to estimate the dietary exposure to Pb and Cd from eating eggs. Table 4 provides a list to the comparison of the results of the present study and others.

**TABLE 4:** Dietary exposure of lead and cadmium through hen eggs consumption in Iran and some studies.

Country	EDI^a^		EWI^b^		EMI^c^		Ref.
	Pb	Cd	Pb	Cd	Pb	Cd	
Bangladesh	0.05	0.06	0.35	0.42	1.5	1.8	Shaheen et al. (2016)
Belgium	0.04	0.0006	0.28	0.0042	1.2	0.018	Waegeneers et al. (2009)
Italy	0.008	0.0014	0.056	0.009	0.24	0.042	Esposito et al. (2016)
Iran	2.7	0.9	18.9	6.3	81	27	Farahani et al. (2015)
Nigeria	0.019	0.002	0.133	0.014	0.57	0.06	Fakayode and Olu‐Owolabi (2003)
Current study	0.002	0.0007	0.014	0.0049	0.06	0.021	–

^a^ Estimated daily intake with the overall concentration of the metals.

^b^ Estimated weekly intake with the overall concentration of the metals.

^c^ Estimated monthly intake with the overall concentration of the metals.

The risk values (PTWI and PTMI) depend on the amount of consumption, the pollution rate of the desired food, and the weight of the target group. The PTWI of Pb and Cd was established at 25 and 7 μg kg^−1^ BW/week by JECFA (FAO/WHO, 2004).

Regarding the overall concentration of Pb in eggs in the present study (0.007 mg kg^−l^), the weekly intake of Pb for adults through eggs consumption was estimated to be 0.014 mg/week which was higher than those reported by (Zheng et al., 2007; 0.0052 mg/week) in China and below those reported by Waegeneers N et al. (0.28 mg/week) in Belgium (Waegeneers et al., 2009), Esposito M et al. (0.056 mg/week) in Italy (Esposito et al., 2016), and Shaheen N et al (0.35 mg/week) in Bangladesh (Shaheen et al., 2016).

The weekly intake of Cd from ingested eggs for adults (in the maximum scenario) was 0.0049 mg/week (0.0007 mg/day). In Italy, reported by Esposito M et al (0.009 mg/week; Esposito et al., 2016), Waegeneers N et al. (0.004 mg/week) in Belgium (Waegeneers et al., 2009), and Fakayode SO et al. (0.014 mg/week) in Nigeria, but was below those reported Farahani S et al. (6.3 mg/week) in Iran (Farahani et al., 2015), which seems that compared with the data of the current study, there has been a decreasing trend in Iran (Table 4).

Due to its long half‐life, the JECFA declared in 2010 that daily cadmium ingestion through food consumption has very little impact on overall exposure. As a result, the acceptable and dietary intake of Cd should be assessed over a month or several months, respectively, to assess the long‐term and short‐term health concerns. Therefore, the JECFA recommended using PTMI instead of PTWI for this dangerous metal, with a value of 25 g kg^−1^ BW/mount (FAO/WHO, 2010) Therefore, Iranian adults should not consume more than 1750 g of cadmium every month. When taking into account the maximum Cd content in eggs, the EMI for adults was 54.43 g/month. Although the exposure to Pb and Cd from eating eggs (EWI and EMI) in the current study was lower than the risk values predicted, this still suggests a little risk for consumers. It should be noted that this study solely covers egg consumption, which may have a very minor impact on Iranian consumers' overall exposure to Pb and Cd. (JECFA, 2011).

### 3.3 Non‐carcinogenic risk evaluation

**FIGURE 1:**
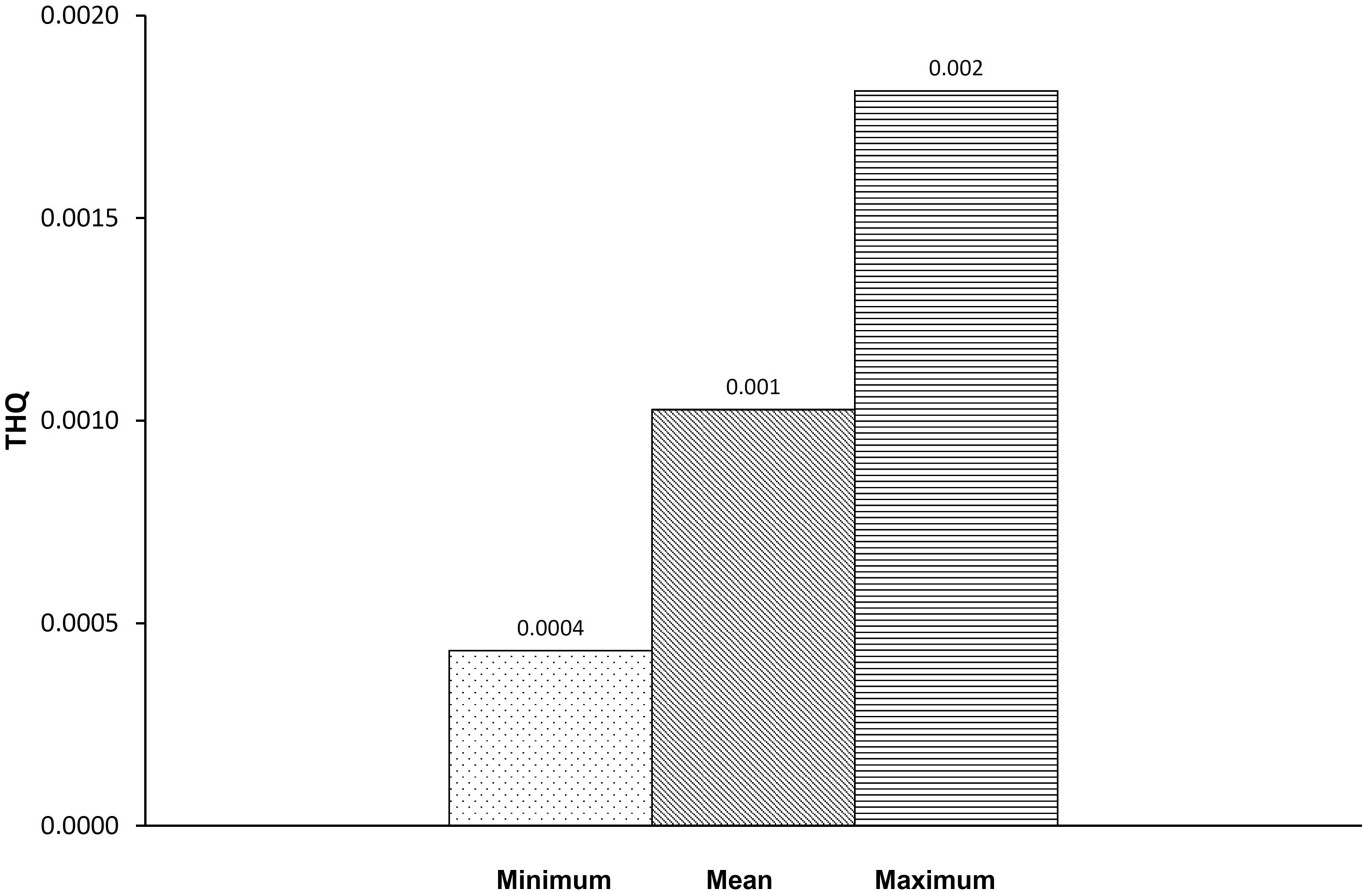
Target hazard quotient (THQ) of Cadmium (Cd) through hen eggs consumption.

**FIGURE 2:**
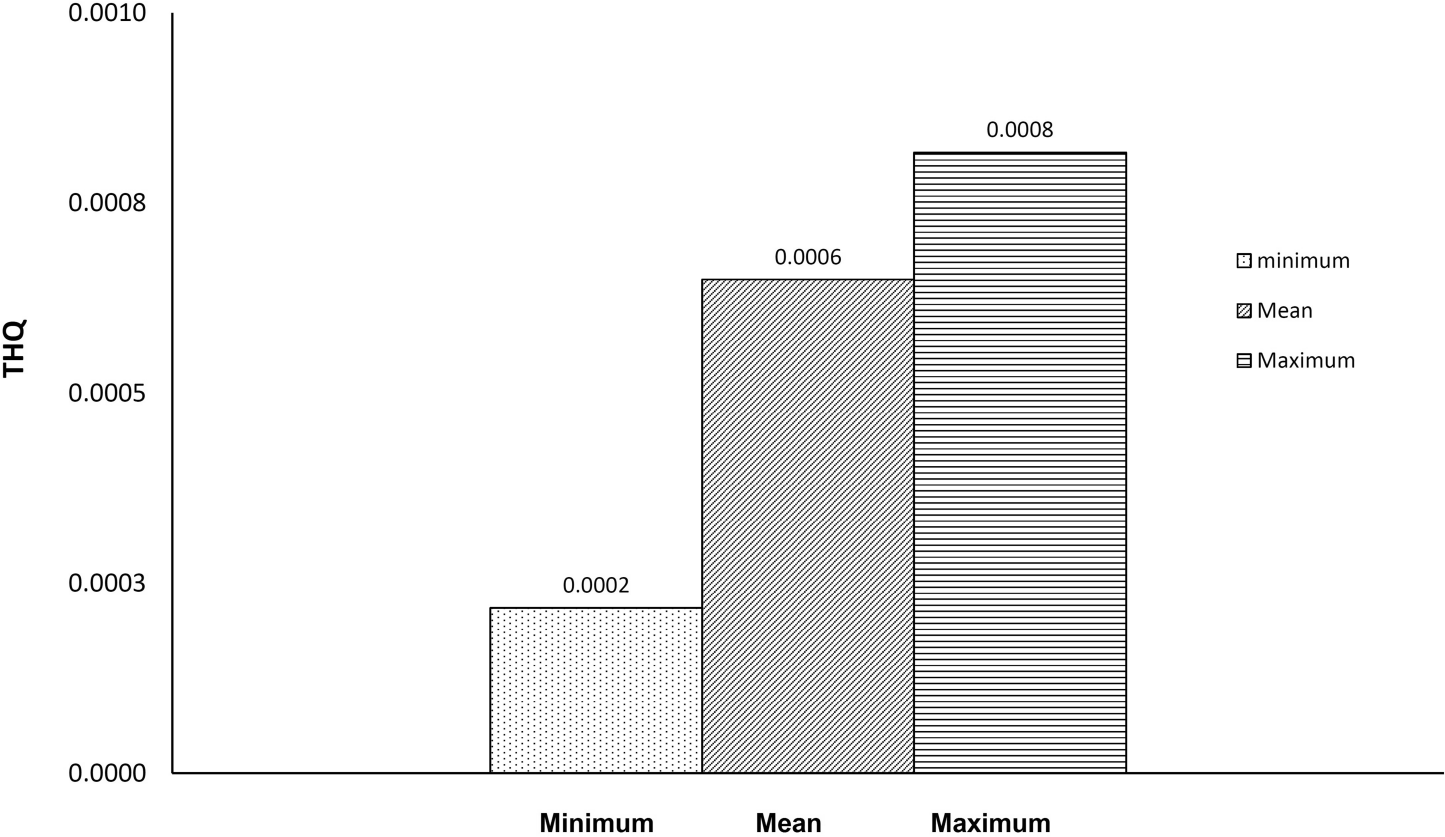
Target hazard quotient (THQ) of Lead (Pb) through hen eggs consumption.

By estimating the THQ (target hazard quotient) value, the non‐carcinogenic danger of lead and cadmium in consumers was determined. The findings of the ICP‐MS analysis were used to determine the total and maximum values of Pb and Cd for adults, as shown in Figures 1 and 2. This value has been accepted as a suitable variable for evaluating the risks connected to consuming dangerous metals through tainted food.

The THQ is defined as a ratio of a contaminant's measured level to a substance's reference oral dose (RFD; Meshref et al., 2014) When a metal's THQ is greater than 1, negative effects are possible; however, when is less than 1, detrimental effects are unlikely to occur (Dadar et al., 2017; Fakhri et al., 2018; Rahmani et al., 2018).

Based on the total amount of eggs consumed daily by Iranian adults (25.4 g/day; NNFTRI, 2022), the THQ of Pb for adults was calculated to be 0.0006, which was lower than China (0.009; Zheng et al., 2007), Egypt (0.005; Hashish et al., 2012), Turkey (0.005; Arslanbaş & Baydan, 2013), and Bangladesh (0.008; Shaheen et al., 2016); on the contrary, Hungary (0.107; Atamaleki et al., 2020) and Poland (1.280; Atamaleki et al., 2020) were the highest in THQ values. In contrast to Turkey, the THQ value for adults in the current study for Cd was 0.001. (0.387; Arslanbaş & Baydan, 2013), while the majority of the results of the examined other studies were similar to ours (Table 5).

**TABLE 5:** THQ of Pb and Cd in adults due to ingestion of eggs in various countries.

Countries	Pb	Cd	Ref.
Bangladesh	0.008	–	Shaheen et al. (2016)
Belgium	0.012	0.003	Waegeneers et al. (2009)
Egypt	0.005	–	Hashish et al. (2012)
France	0.011	0.002	Malmauret et al. (2002)
Hungary	0.107	–	Atamaleki et al. (2020)
India	0.021	–	Giri and Singh (2019)
Italy	0.024	0.009	Esposito et al. (2016)
Kuwait	0.018	0.006	Husain et al. (1996)
Nigeria	0.035	0.014	Fakayode and Olu‐Owolabi (2003)
Pakistan	0.023	–	Khan et al. (2014)
Palestine	0.035	0.017	Abdulkhaliq et al. (2012)
Poland	1.280	0.080	Atamaleki et al. (2020)
Thailand	0.515	–	Tulayakul et al. (2018)
Turkey	0.005	0.387	Arslanbaş and Baydan (2013)
China	0.009	0.002	Zheng et al. (2007)
Current study	0.0006	0.001	–

Lead builds up over time in bones and soft tissue, but more significantly, its presence in blood and bones over its half‐life has been demonstrated. The cardiovascular effects and developmental neurotoxicity in adults and young children were acknowledged by the JECFA and CONTAM Panel of the EFSA (European Food Safety Authority) as being significant for the assessment of lead risk (Abadin et al., 2007; Norouzirad et al., 2018). Due to an interaction with a protein with a high sulfhydryl content, the majority of the absorbed Cd is stored in the kidneys. The half‐life of Cd in human kidneys could be up to 30 years. Emphysema, chronic obstructive lung disease, and chronic renal tubular disease are the main outcomes of a long‐term cadmium buildup, particularly in children (Faroon et al., 2012; Friberg, 2017). Adult THQ values from consuming eggs have been reported by several authors and are displayed in Table 5. For example (Gonzalez et al., 2017) reported values of 0.035 for Pb, which is equal to that discovered in this study, and 0.045 for Cd, which is greater than that obtained in this work. These results were published in Mexico. Pb and Cd concentrations were discovered by (Zheng et al., 2007) in China to be 0.009 and 0.002, respectively, lower than those detected in our investigation. In Pakistan, Khan et al. (2014) reported Pb values of 0.023. According to the result of this study, eating eggs poses no health risks to Iranian consumers, despite the THQ values of Pb and Cd for adult consumers in this research showing values below one (even in the worst‐case scenario). It is important to note that exposure to these toxic metals from sources other than those examined in the study, such as water, skin contact, inhalation of dust, and consumption of other foods, may result in THQ values greater than 1, which would indicate a significant risk to the health of the exposed population.

### 3.4 Carcinogenic risk

Numerous studies have demonstrated that exposure to environmental contaminants, such as toxic elements, increases the risk of cancer, although a range of factors, including age, race, and gender, may contribute to the development of cancer (Antwi et al., 2015; Farokhi et al., 2017; Steenland & Boffetta, 2000).

**FIGURE 3:**
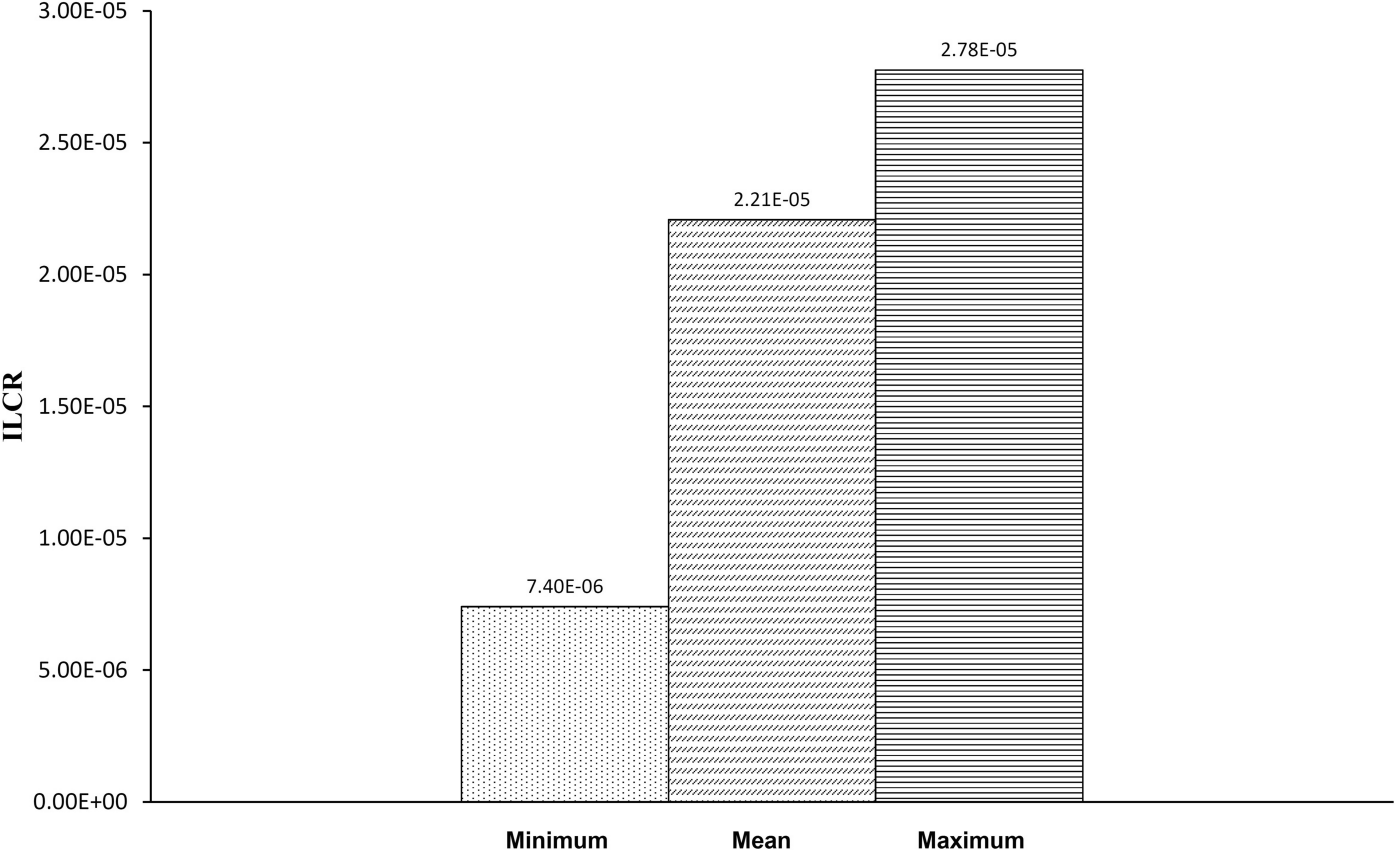
Incremental lifetime cancer risk (ILCR) of Lead (Pb) through hen eggs consumption.

IARC has identified Pb and Cd as possible contributors to the development of human cancer, as was previously mentioned (IARC, 1993, 2006, 2017). To calculate the carcinogenic risk for adult egg users in the current investigation, the ILCR value was used. Because the CSF for cadmium's risk of oral cancer has not yet been established, we only estimated the ILCR for lead. (Figure 3). Incremental lifetime cancer risks for Pb were found to be 2.2E‐05 and 2.8E‐05, respectively. The U.S. Environmental Protection Agency (US‐EPA) states that the safe limit for cancer risk is below approximately 1 chance in 1000,000 lifetime exposure (ILCR < 10^−6^), the threshold risk limit (ILCR > 10^−4^) for a chance of cancer is above 1 in 10,000 exposure, where corrective measures are significant, and the moderate risk level (ILCR > 10^−3^) is above 1 in 1000 where public health safety assessment is more critical (Pepper et al., 2012; Tchounwou et al., 2012). The findings showed that the adult group's consumption of eggs did not increase their risk of developing cancer and that their health is almost safe at this point (10^−4^ > ILCR < 10^−6^).

**FIGURE 4:**
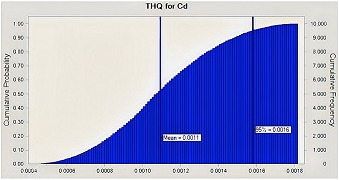
Target hazard quotient (THQ) distribution for Cd through hen eggs consumption by Monte Carlo simulation.

**FIGURE 5:**
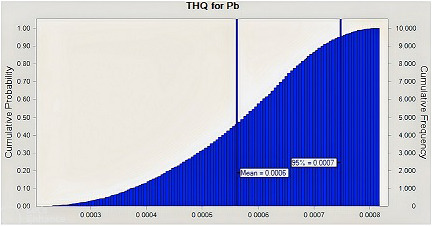
Target hazard quotient (THQ) distribution for Lead (Pb) through hen eggs consumption by Monte Carlo simulation.

**FIGURE 6:**
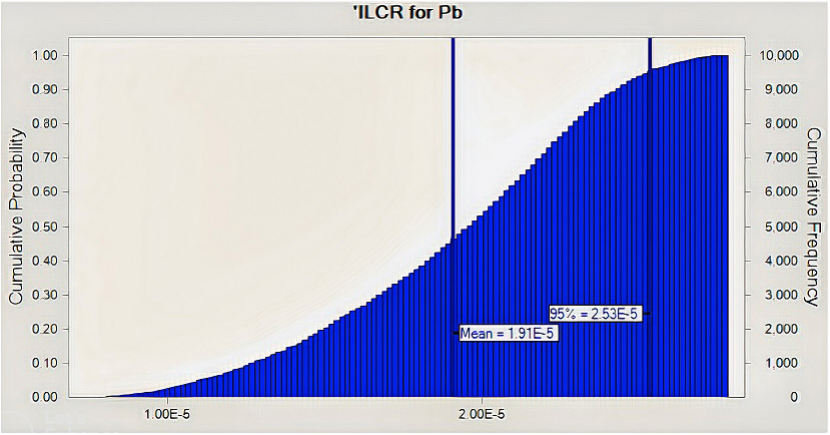
Incremental lifetime cancer risk (ILCR) distribution for Lead (Pb) through hen eggs consumption by Monte Carlo simulation.

When calculating health hazards, there may be some uncertainty (Chen et al., 2012). High uncertainty is seen when single‐point data are used to determine health risks associated with exposure to contaminants such as hazardous metals. As a result, MCS was employed in our study as a probabilistic technique to reduce the uncertainties in the health risk evaluation. According to the MCS, the estimated THQ values for Pd and Cd for adults at the 95% percentile were 0.0007 and 0.002, respectively (Figures 4 and 5) both of which were <1, thus demonstrating that Iranian consumers are not potentially at risk for health problems as a result of consuming eggs. Additionally, the ILCR of Pb for adults was found to be 2.5E‐05, which did not cross the cancer risk threshold (Figure 6).

Through contaminated nutrition, dirty water sources, and additional parameters including age, species, and lying cycle, these substances are introduced in hen eggs. Supplemental nutrition and insecticides used to get rid of insects are other factors that affect hen eggs (Waegeneers et al., 2009). It should come as no surprise that chicken farms require oversight, particularly in industrialized regions.

Our food chain is constantly being replenished with essential and non‐essential materials as a result of the excessive use of agrochemicals, municipal wastewater, industrial effluents, and raw sewage for (Tongesayi et al., 2013).

### 3.5 Recent approaches

The findings of studies carried out in Iran indicate that the health risks (whether cancerous or not) of the heavy metals examined in Iranian egg products are safe for a variety of consumers and that no intervention or remediation is required to lessen the contamination burden of heavy metals in these poultry sectors. Increased research on metal contamination of food, especially eggs, is a result of environmental pollution from trace elements nowadays. Eggs are a good indicator of environmental pollution (Esposito et al., 2016; Tongesayi et al., 2013). In another word, the presence of heavy metal and trace element residues in chicken eggs may indicate chemical risks from the perspectives of the feed safety sector owing to ingestion of contaminated feeds or it is partially responsible for the poultry medicine sector. (Saad Eldin & Raslan, 2018). Thus, hen eggs can absorb heavy metals from different sources in the environment and transmit them into their eggs (Aendo et al., 2018).

In addition, it might have presented difficulties and contributed to the chickens' worse health as a result of prolonged exposure. It is required to conduct more research by ongoing monitoring of the production of hen eggs contaminated with heavy metals. To control and reduce the intake of heavy metals, more consideration should be given to this issue. It is advised to raise stakeholders' awareness, such as farmers, in industrial settings. Additionally, monitoring is a secure strategy to eliminate lead and cadmium.

### 3.6 Limitation

It is recommended that the international establishes this restriction in response to the requirements of the food safety authorities because there are no trustworthy standard limits for Pb and Cd in hen eggs. It is advised that future studies evaluate the presence of heavy metals in chicken feed, water, and meat on an individual basis. Due to lockdown and budget limitations, our samples may not be reprehensive of all eggs in Iran. Moreover, this study represents the heavy metals content in eggs and does not necessarily reflect other product consumption.

## 4 CONCLUSION

An appropriate and sensitive ICP‐MS method was used to evaluate the levels of lead and cadmium in hen egg samples in Iran. The findings of the present investigation showed that lead and cadmium levels in all evaluated eggs were suitable for human consumption. Adults' Pb and Cd exposure from eating eggs was significantly lower than the risk levels established by JECFA, per the exposure assessment. According to the THQ values of these dangerous metals, which were below one value, egg eating by Iranian consumers does not present a non‐carcinogenic risk. Due to the Pb concentration in eggs, the carcinogenic risk assessment concluded that adults in Iran are not at increased risk of developing cancer (ILCR > 10^−6^). However, other meals containing these heavy metals can also introduce lead and cadmium into the body. As a result, it is advised that lead and cadmium levels in hen eggs and other foods in Iran be routinely monitored.

## FUNDING INFORMATION

National Nutrition and Food Technology Research Institute, Faculty of Nutrition Sciences and Food Technology, Shahid Beheshti University of Medical Sciences, Tehran, Iran (Grant No: 30109).

## CONFLICT OF INTEREST STATEMENT

The authors declare that they have no conflicts of interest to disclose.

## ETHICAL APPROVAL

The Ethics Committee of the National Nutrition and Food Technology Research Institute, Faculty of Nutrition Science and Food Technology, Shahid Beheshti University of Medical Sciences, approved the project by ethical code: IR.SBMU.nnftri.Rec.1400.074. This article does not contain any studies involving human participants performed by any of the authors. Also, it does not contain any studies involving animals performed by any of the authors.

## Data Availability

The data will be available upon request from the corresponding authors.
